# Draft Genome Sequence of the Thermotolerant Cyanobacterium *Desertifilum* sp. IPPAS B-1220

**DOI:** 10.1128/genomeA.01304-16

**Published:** 2016-11-17

**Authors:** Kirill S. Mironov, Maria A. Sinetova, Kenzhegul Bolatkhan, Bolatkhan K. Zayadan, Vera V. Ustinova, Elena V. Kupriyanova, Alexandra N. Skrypnik, Natalya E. Gogoleva, Yuriy V. Gogolev, Dmitry A. Los

**Affiliations:** a Institute of Plant Physiology, Russian Academy of Science, Moscow, Russia; b Department of Biotechnology, Faculty of Biology and Biotechnology, Al-Farabi Kazakh National University, Almaty, Kazakhstan; c Microbiology Department, Central Tuberculosis Research Institute RAMS, Moscow, Russia; d Kazan Institute of Biochemistry and Biophysics, Kazan Science Centre, Russian Academy of Sciences, Kazan, Russia

## Abstract

Here, we report the draft genome of the filamentous cyanobacterium *Desertifilum* sp. strain IPPAS B-1220, isolated from Lake Shar-Nuur, Mongolia. The genome of 6.1 Mb codes for 5,113 genes. Genome mining revealed 10 clusters for the synthesis of bioactive compounds (nonribosomal peptides, polyketides, bacteriocins, and lantipeptides) with potential biotechnological or medical importance.

## GENOME ANNOUNCEMENT

Cyanobacteria produce a broad range of secondary metabolites with diverse chemical structures. The majority of these metabolites are the products of nonribosomal peptide synthetase or polyketide synthase pathways (1). We sequenced the genome of the cyanobacterium *Desertifilum* sp. strain IPPAS B-1220 (family, *Oscillatoriaceae*), which was newly isolated from the freshwater Lake Shar-Nuur, Mongolia.

Genomic DNA was isolated from cells grown to stationary phase by incubation with saturated iodide solution followed by lysozyme treatment and 2% SDS lysis at 70°C (2). DNA was purified by phenol-chloroform extraction. Isolated DNA was fragmented by adaptive focused acoustics technology using a Covaris S220 ultrasonicator (Covaris, Woburn, MA, USA). Parameters for DNA shearing were adapted to obtain 500-bp fragments. A DNA library was prepared with a NEBNext Ultra DNA library prep kit (NEB, Ipswich, MA, USA) for Illumina sequencing, which was performed on the MiSeq system with MiSeq reagent kit version 3 in a 600-cycle paired-end format. The sequence quality was analyzed by FastQC (http://www.bioinformatics.babraham.ac.uk/projects/fastqc). Adapters and low-quality nucleotides were trimmed with Trimmomatic (3). The genome was assembled using AbySS (4) and SPAdes (5) software. The quality of the draft genome assembly was analyzed by QUAST (6). The draft genome coverage was no less than 30×, with *N*_50_ statistics >10^5^. The approximate genome size is 6.1 Mb, with an estimated average G+C content of 48.7%.

The genome was annotated using the automated NCBI Prokaryotic Genome Annotation Pipeline (PGAP). It contains 5,113 genes: 4,964 genes coding for proteins, 86 pseudogenes, six rRNA-coding sequences (four for 5S, one for 16S, and one for 23S), 53 tRNAs, and four noncoding RNAs. Seven CRISPR arrays were found in the genome. The genome sequence was analyzed with the antiSMASH tool (7) for the presence of gene clusters coding for secondary metabolite biochemical pathways. Eight contigs contained gene clusters for secondary metabolite biosynthesis (nonribosomal peptides, polyketides, bacteriocins, lantipeptides, etc.). Thus, genome mining of *Desertifilum* sp. IPPAS B-1220 revealed a set of putative bioactive compounds that may have further biotechnological or pharmaceutical applications.

### Accession number(s).

The genome sequence of *Desertifilum* sp. strain IPPAS B-1220 was deposited at NCBI with the following attributes: SAMN05788062 for BioSample, PRJNA343432 for BioProject, and SRR4255595 for the SRA database. The genome PGAP file accession number is MJGC00000000.
